# Area asymmetry of heart rate variability signal

**DOI:** 10.1186/s12938-017-0402-3

**Published:** 2017-09-21

**Authors:** Chang Yan, Peng Li, Lizhen Ji, Lianke Yao, Chandan Karmakar, Changchun Liu

**Affiliations:** 1 School of Control Science and Engineering, Shandong University, Jinan, 250061 China; 2 School of Information Technology, Deakin University, Burwood, VIC 3125 Australia

**Keywords:** Heart rate asymmetry (HRA), Heart rate variability (HRV), Poincaré plot, Area asymmetry, Phase asymmetry

## Abstract

**Background:**

Heart rate fluctuates beat-by-beat asymmetrically which is known as heart rate asymmetry (HRA). It is challenging to assess HRA robustly based on short-term heartbeat interval series.

**Method:**

An area index (AI) was developed that combines the distance and phase angle information of points in the Poincaré plot. To test its performance, the AI was used to classify subjects with: (i) arrhythmia, and (ii) congestive heart failure, from the corresponding healthy controls. For comparison, the existing Porta’s index (PI), Guzik’s index (GI), and slope index (SI) were calculated. To test the effect of data length, we performed the analyses separately using long-term heartbeat interval series (derived from >3.6-h ECG) and short-term segments (with length of 500 intervals). A second short-term analysis was further carried out on series extracted from 5-min ECG.

**Results:**

For long-term data, SI showed acceptable performance for both tasks, i.e., for task i *p* < 0.001, Cohen’s *d* = 0.93, AUC (area under the receiver-operating characteristic curve) = 0.86; for task ii *p* < 0.001, *d* = 0.88, AUC = 0.75. AI performed well for task ii (*p* < 0.001, *d* = 1.0, AUC = 0.78); for task i, though the difference was statistically significant (*p* < 0.001, AUC = 0.76), the effect size was small (*d* = 0.11). PI and GI failed in both tasks (*p* > 0.05, *d* < 0.4, AUC < 0.7 for all). However, for short-term segments, AI indicated better distinguishability for both tasks, i.e., for task i, *p* < 0.001, *d* = 0.71, AUC = 0.71; for task ii, *p* < 0.001, *d* = 0.93, AUC = 0.74. The rest three measures all failed with small effect sizes and AUC values (*d* < 0.5, AUC < 0.7 for all) although the difference in SI for task i was statistically significant (*p* < 0.001). Besides, AI displayed smaller variations across different short-term segments, indicating more robust performance. Results from the second short-term analysis were in keeping with those findings.

**Conclusion:**

The proposed AI indicated better performance especially for short-term heartbeat interval data, suggesting potential in the ambulatory application of cardiovascular monitoring.

**Electronic supplementary material:**

The online version of this article (doi:10.1186/s12938-017-0402-3) contains supplementary material, which is available to authorized users.

## Background

Heart rate variability (HRV), commonly indicated by the variation within the time interval between heartbeats, has been intensively studied for several decades [1, 2]. It comes from the spontaneity of the sinoatrial (SA) node as well as the adaptability or resilience of SA node to the stimuli of both internal control system (e.g., autonomic nervous system) and external ever-changing environment [3, 4]. In other words, HRV may help understand how well the underlying control mechanism works and further, provides an opportunity to capture the tiny but early signs that may predict the future cardiovascular risk in healthy individuals, for whom invasive and costly assessments are usually infeasible.

Various methods have been established for assessing HRV [5, 6]. However, increasing efforts are most recently made to develop those that can understand the dynamics of heart rate (HR) [7, 8], e.g., Poincaré plot which is able to not only visualize but also quantitatively evaluate the oscillatory patterns of HRV [6, 9–18]. The Poincaré plot draws the current RR interval against the preceding one [15, 19], resulting in a scatter diagram which visualizes both the increase and decrease of HR. It thus may imply important information about the balance between sympathetic and parasympathetic activities. Previous studies have indicated that the Poincaré plot is physiologically asymmetric with respect to line of identity (the line on which the current heartbeat interval is identical to the preceding one) [14, 15] and this asymmetry changes in diseases, e.g., arrhythmia [19], heart failure [16], obstructive sleep apnea [20], myocardial infarction [21, 22], postoperative myocardial ischemia [23], and type 1 diabetes [24], etc., suggesting possibly an imbalance in autonomic control under those pathological conditions.

Several measures have been developed to assess the asymmetry of Poincaré plot (or termed as heart rate asymmetry, HRA), e.g., Porta’s index (PI) evaluates the asymmetry of the amounts of points located in two regions of Poincaré plot separated by the line of identity [11], Guzik’s index (GI) measures the asymmetry based on the cumulative distance of each point to the line of identity [9], and slope index (SI) quantifies the asymmetry of the phase angles [19]. In this study, we proposed that the asymmetry could also be assessed by combining the distance and phase angle characteristics. Moreover, by combining these two pieces of information, HRA could be better assessed for short-term HRV series than existing indices. To test these two hypotheses, we first developed an area index (AI) which exactly combined the two characteristics—distance and phase angle. We then compared the performance of AI with previously established PI, GI, and SI in classifying arrhythmia from normal sinus rhythm, as well as in classifying patients with congestive heart failure from healthy control subjects based on long-term (derived from >3.6-h ECG recordings) and short-term (500 RR intervals) HRV series, separately. In a third analysis, we applied the HRA analyses to short-term heartbeat interval series extracted from 5-min ECG of the patients with congestive heart failure and healthy subjects from our clinical trials.

## Method

### Area index (AI)

**Fig. 1:**
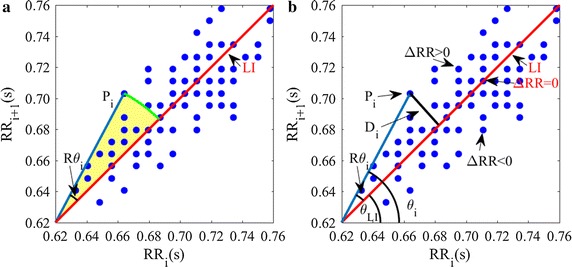
Schematic representation of heart rate asymmetry algorithms. Blue dots show points in the Poincaré plot. *P*_*i*_ is the *i*th point which can be denoted by $(RR_{i} ,RR_{i + 1} ),\Delta RR = RR_{i + 1} - RR_{i}$. LI is the line of identity. **a** $R\theta_{i}$ is the phase angle of the *P*_*i*_ respect to LI. Filled areas are the sectors we used in AI; **b**. *D*_*i*_ is the distance of *P*_*i*_ to LI and $\theta_{i}$ the phase angle of *P*_*i*_ respect to the abscissa; $\theta_{LI}$ is the phase angle of LI

AI is defined as the cumulative area of the sectors corresponding to the points that are located above the line of identity (LI) divided by the cumulative area of sectors corresponding to all points in the Poincaré plot (except those that are located exactly on LI). Specifically, a certain point $P_{i}$ in the Poincaré plot can be denoted by $(RR_{i} ,RR_{i + 1} )$ if the HRV series is defined as $(RR_{1} ,RR_{2} , \ldots ,RR_{i} ,RR_{i + 1} , \ldots ,RR_{n} )$. The area of the sector corresponding to point $P_{i}$, as shown schematically in Fig. 1a, can be calculated by

1 $$ S_{i} = \frac{1}{2} \times R\theta_{i} \times r^{2} $$

wherein, *r* is the radius of the sector; $R\theta_{i} = \theta_{LI} - \theta_{i}$; $\theta_{LI}$ is the phase angle of LI, and $\theta_{i}$ is the phase angle of the *i*-th point which is defined as $\theta_{i} = atan\left( {\frac{{RR_{i + 1} }}{{RR_{i} }}} \right)$. Then, AI can be calculated by.

2 $$ AI = \frac{{\sum\nolimits_{i = 1}^{l} {\left| {S_{i} } \right|} }}{{\sum\nolimits_{i = 1}^{m} {\left| {S_{i} } \right|} }} \times 100 $$

wherein, *l* means the number of points above LI and *m* means the number of points which are not on LI (*m* = *l* + *b* wherein *b* is the number of points below LI).

### Existing indices

#### Porta’s index (PI)

PI was defined as the number of points below LI divided by the total number of points in Poincaré plot except those that are located on LI, specifically,

3 $$ PI = \frac{b}{m} \times 100 $$

wherein, *b* is the number of points below LI and *m* is the total number of points except those that are not on LI.

#### Guzik’s index (GI)

GI was defined as the distance of points above LI to LI divided by the distance of all points in Poincaré plot to LI except those that are located on LI. Specifically,

4 $$ GI = \frac{{\sum\nolimits_{i = 1}^{l} {D_{i} } }}{{\sum\nolimits_{i = 1}^{m} {D_{i} } }} \times 100 $$

wherein, *l* means the number of point above LI; *m* means the number of points in Poincaré plot except those which are not on LI; *D*_*i*_ is the distance of point *P*_*i*_ to LI which can be calculated using

5 $$ D_{i} = \frac{{\left| {RR_{i + 1} - RR_{i} } \right|}}{\sqrt 2 } $$

#### Slope index (SI)

SI was defined as the phase angle of points above LI divided by the phase angle of all points in Poincaré plot except those that are not located on LI. SI can be calculated using

6 $$ SI = \frac{{\sum\nolimits_{i = 1}^{l} {\left| {R\theta_{i} } \right|} }}{{\sum\nolimits_{i = 1}^{m} {\left| {R\theta_{i} } \right|} }} \times 100 $$

wherein, *l* means the number of point above LI and *m* means the number of all points except those that are not on LI.

Figure 1b shows schematically the ideas behind PI, GI, and SI.

### Materials

Data used in this study were from the PhysioBank archives [25] and a previous clinical trial we performed. Specifically, based on PhysioBank database, we proposed to perform two case studies: one was between the HRV of healthy subjects (group HEA) and HRV of arrhythmia subjects (group ARR); the other one was between the HEA group and HRV of patients with congestive heart failure (group CHF).

The HEA group consists of 54 HRV series of healthy subjects (age: 61.4 ± 11.6 [mean ± SD unless otherwise indicated], 30 men and 24 women) from the normal sinus rhythm RR interval database [25]. The original ECG data for them, which are available in another database (MIT-BIH normal sinus rhythm database), were digitized at a sampling frequency of 128 Hz. RR intervals were calculated based on the incorporated beat annotation files which were obtained by automated analysis with manual review and correction [25].

For the ARR and CHF groups, the CAST RR interval sub-study database and congestive heart failure RR interval database were used, respectively. The former one contains three different sub-sets (corresponding to interventions using three different medications), among which we only used the Encainide group in order to be comparable with a previous study [19]. This sub-set consists of 272 subjects (age: 60.0 ± 10.1, 227 men and 45 women) who had an acute myocardial infarction (MI) within preceding 2 years and have at least six premature ventricular contractions (PVCs) per hour according to 24 h Holter ECG (pre-treatment). Those subjects enrolled within 90 days of the index MI were required to have left ventricular ejection fractions less than or equal to 55%, while those enrolled after this 90-day window were required to have an ejection fraction less than or equal to 40%. The congestive heart failure RR interval database includes 29 RR intervals from patients with congestive heart failure (age: 55.3 ± 11.6, 8 men and 2 women, gender unknown for the rest 19 subjects, New York Heart Association [NYHA] class I 4 subjects, II 8 subjects, and III 17 subjects). Similar to the HEA group, the original ECG recordings for both the ARR and CHF groups were sampled at 128 Hz and the beat annotation files were used to calculate the RR interval [25].

In a third case study, we were still testing the performance on short-term heartbeat interval series but this time the actual lengths of the series were different. Instead, they were extracted from ECG data of the same length, i.e., 5-min. The data came from one of our previous clinical trials [8, 26–29]. 28 CHF patients (age: 59.7 ± 9.8, 16 men and 12 women) and 29 age- and sex-matched healthy volunteers (age: 59.0 ± 6.9, 15 men and 14 women) were recruited and provided informed consents in that study. The HF patients were in NYHA class II–III with functional classification confirmed by the ultrasonic cardiogram. Measurements were undertaken in a quiet, temperature-controlled clinical measurement room (25 ± 3 °C) at Qilu Hospital of Shandong University, by a cardiovascular function detection device (CV FD-I) produced by Huiyironggong Technology Co., Ltd, Jinan, China. ECG data in standard limb II configuration were recorded in supine position for 5 min at a sampling frequency of 1 kHz after a 10-min rest. All study procedures were approved by the Clinical Ethics Committee of the Qilu Hospital of Shandong University and were conducted according to the principles in the Declaration of Helsinki. R peaks were detected through a template matching procedure and ectopic R peaks were identified [29]. RR interval series were constructed by the intervals of consecutive normal R peaks.

### HRA analyses

HRA was assessed by the proposed AI and the three previously established indices. Note that before obtaining the Poincaré plots, the raw HRV series were preprocessed by subtracting the corresponding minimum RR interval. In order to understand the performance in handling both long-term and short-term HRV series, three analyzing protocols on the three databases from PhysioNet were applied: (a) using the complete HRV series (corresponding to 3.6–25.95-h ECG recordings, mean ± SD: 22.45 ± 2.08 h); (b) using short-term HRV segment (with a length of 500 RR intervals, corresponding to ~5 min ECG recordings) randomly selected from the complete series; (c) using 10 subsequent short-term HRV segments (500 intervals) with a 50% overlap between each two selected from the very beginning of each HRV series. To estimate the HRA of the clinical data, the four indices were applied to the short-term heartbeat interval data (obtained from 5 min ECG recordings) directly.

## Statistical analyses

The between-group difference (i.e., HEA vs. ARR, HEA vs. CHF, and healthy volunteers vs. CHF) of each specific HRA index was analyzed by the non-parametric Mann–Whitney *U* test, with the effect size estimated by Cohen’s *d*. Classification performance was further examined using the area under the receiver-operating characteristic curve (ROC). Statistical significance was accepted at *p* < 0.05. A smaller effect size was considered if *d* < 0.5 [30, 31]. All statistical analyses were performed using the SPSS software (version 23, IBM, New York, USA).

## Results

### HRA assessed using the complete HRV series (analysis protocol a)

**Fig. 2:**
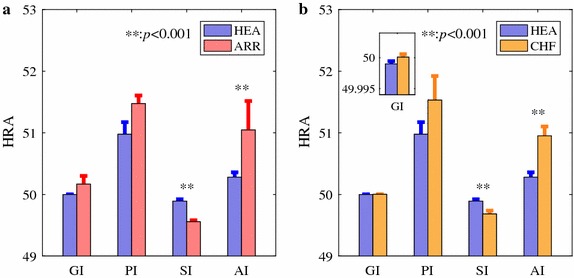
HRA results estimated by GI, PI, SI, and AI. **a** A comparison between healthy subjects and subjects with arrhythmia (results obtained from 54 healthy subjects and 272 subjects with arrhythmia). **b** A comparison between healthy subjects and CHF patients (results obtained from 54 healthy subjects and 29 CHF patients). Bar indicates mean level and error bar the standard error (SE). All HRA indices were calculated based on the complete HRV recordings. **p <0.001

HRA results assessed by all four indices (PI, GI, SI, and AI) calculated using the complete HRV series are summarized as mean + standard error (SE) in Fig. 2. No statistically significant difference was indicated between HEA and ARR groups for both PI and GI, whereas both SI and AI suggested that the HRV was more asymmetric in ARR group than that in HEA group (*p* > 0.1 for PI and GI, *p* < 0.001 for SI and AI), as shown in Fig. 2a. Note that any value of HRA that deviates from 50% should suggest asymmetric. Depending on which side (above the LI or below it) weights higher, the value can be either smaller or larger than 50%. Besides, larger absolute deviations from 50% suggest stronger asymmetry. However, only SI showed a large effect size (*d* = 0.9), whereas *d* < 0.3 for the other three indices. For the comparison between HEA and CHF groups, PI and GI did not show statistically significant difference (both *p* > 0.05, *d* < 0.4) whereas SI and AI indicated more asymmetric HRV in CHF group (both *p* < 0.001; *d* = 0.8 and 1.0, respectively for SI and AI), as shown in Fig. 2b.

**Fig. 3:**
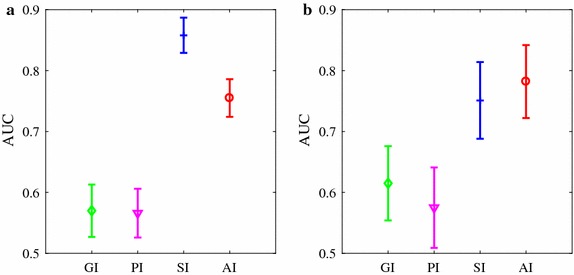
AUC results of the four HRA indices. **a** A comparison between healthy subjects and subjects with arrhythmia (results obtained from 54 healthy subjects and 272 subjects with arrhythmia). **b** A comparison between healthy subjects and CHF patients (results obtained from 54 healthy subjects and 29 CHF patients). Results were based on the complete HRV recordings and are shown as mean ± stardard error (SE, nonparametric bootstrap-based estimator in SPSS)

Figure 3 shows the area under ROC (AUC) ± SE (standard error) of the four indices for classifying ARR from HEA groups, as well as classifying CHF group from HEA group. Similarly, acceptable AUC values were obtained by SI and AI (ARR vs. HEA: 0.86 and 0.76, respectively for SI and AI; CHF vs. HEA: 0.75 and 0.78, respectively) while AUC < 0.65 for PI and GI in both comparisons. The ROC plots are provided in the Additional file 1.

### HRA assessed using short-term HRV series (analysis protocols b and c)

**Fig. 4:**
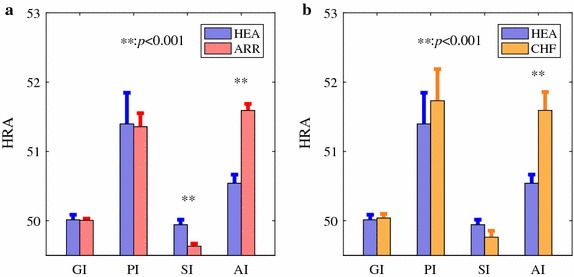
HRA results estimated by GI, PI, SI, and AI. **a** A comparison between healthy subjects and subjects with arrhythmia (results obtained from 54 healthy subjects and 272 subjects with arrhythmia). **b** A comparison between healthy subjects and CHF patients (results obtained from 54 healthy subjects and 29 CHF patients). Bar indicates mean level and error bar the standard error (SE). All HRA indices were calculated based on 500 intervals of HRV recordings. **p <0.001

HRA results assessed by all four indices calculated based on short-term HRV series (500 intervals) are summarized in Fig. 4. No statistically significant difference was indicated between HEA and ARR groups by PI and GI (both *p* > 0.8, *d* < 0.1), whereas SI and AI suggested that the HRV was more asymmetric in ARR group than that in HEA group (both *p* < 0.001; *d* = 0.5 for SI and 0.7 for AI). For the comparison between HEA and CHF groups, PI, GI and SI did not show statistically significant difference (all *p* > 0.2, *d* < 0.4) whereas AI indicated more asymmetry of HRV in CHF group (*p* < 0.001, *d* = 0.9), as shown in Fig. 4b.

**Fig. 5:**
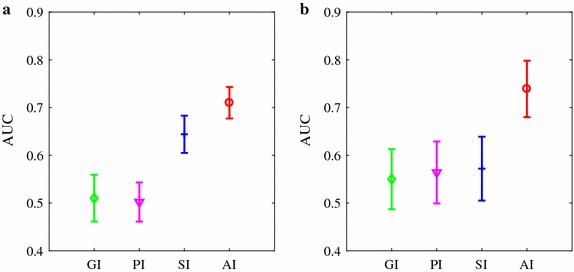
AUC results of the four HRA indices. **a** A comparison between healthy subjects and subjects with arrhythmia (results obtained from 54 healthy subjects and 272 subjects with arrhythmia). **b** A comparison between healthy subjects and CHF patients (results obtained from 54 healthy subjects and 29 CHF patients). Results were based on the 500 intervals of HRV recordings and are shown as mean ± standard error (SE)

Figure 5 shows the AUC ± SE of the four indices for classifying ARR from HEA groups, as well as classifying CHF group from HEA group. Again, acceptable AUC values were only obtained based on AI (AUC = 0.72 and 0.75, respectively, for the two comparisons, AUC <0.6 for PI, GI and SI in both comparisons). The ROC plots are provided in the Additional file 1.

**Fig. 6:**
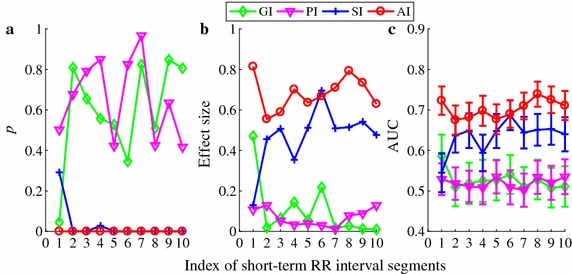
Performance of the four HRA indices (GI, PI, SI, and AI) in differentiating 272 subjects with arrhythmia from 54 healthy subjects. All indices were calculated using 10 different short-term HRV segments. **a** *p* values; **b** effect size *d*; **c** AUC results (shown the mean and error bar the standard error SE)

**Fig. 7:**
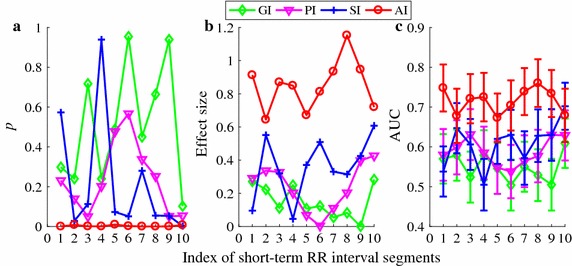
Performance of the four HRA indices (GI, PI, SI, and AI) in differentiating 29 CHF patients from 54 healthy subjects. All indices were calculated using 10 different short-term HRV segments. **a** *p* values; **b** effect size *d*; **c** AUC results (shown the mean and error bar the standard error SE)

Figures 6 and 7 show the *p* values, effect sizes and AUC results for each of the four HRA indices obtained from the 10 short-term HRV segments (see protocol c). Overall, AI showed the best consistency and stability in terms of *p* value, effect size, and AUC. All the other three indices did not suggest statistically significant differences and had small effect sizes for both comparisons except SI which indicated significance for differentiating ARR from HEA for most cases but only occasionally for differentiating CHF from HEA. Besides, the AUC curves for the other three indices were almost all below the AUC curves of AI.

### HRA results of the clinical short-term heartbeat interval data

**Fig. 8:**
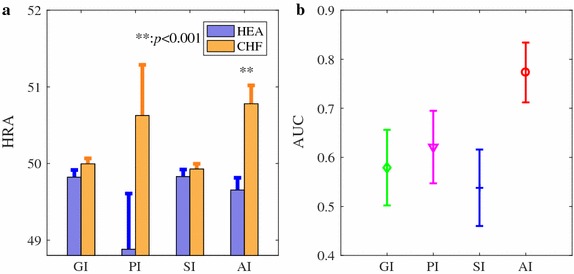
Comparison of HRA estimated by GI, PI, SI, and AI, between 28 CHF patients and 29 healthy volunteers for the clinical data. All HRA indices were calculated based on 5 min ECG recordings. **a** Bar indicates mean of a specific HRA index and error bar standard error (SE). **p < 0.001. **b** AUC results are shown as mean ± stardard error (SE)

Figure 8a summarizes the HRA results of the heartbeat interval series (extracted from 5-min ECG) from our clinical studies. No statistically significant difference in PI, GI and SI (all *p* > 0.1, and *d* < 0.5) was observed between CHF patients and healthy volenteers. However, AI suggested statistically significantly increased asymmetry in CHF patients compared to healthy volunteers (*p* < 0.001, *d* = 1.0). Figure 8b shows the AUC ± SE of the four indices for classifying CHF patients from HEA groups. Only AI indicated relatively larger AUC results (0.77) while AUC <0.7 for all the other three indices. The ROC plots are provided in the Additional file 1.

## Discussion

The heart does not always beat at the same speed. Instead, it accelerates or decelerates over time due to mostly the regulation of autonomic nervous system (ANS) [5, 9, 11, 12, 14, 32–35]. The ANS consists of two branches—sympathetic nervous system, the activation of which speeds up the heartbeat, and parasympathetic nervous system which, when activated, slows down the heartbeat [3, 9, 12, 13, 22, 32, 34, 36]. There is an intrinsic balance between the sympathetic and parasympathetic tones [32]. But this balance does not mean that these two branches contribute equally to heartbeat; instead, the balance may shift to either branch in different conditions and the shift changes from time to time [36, 37] which, consequently, gives rise to an asymmetry between the acceleration and deceleration of heart rate.

**Fig. 9:**
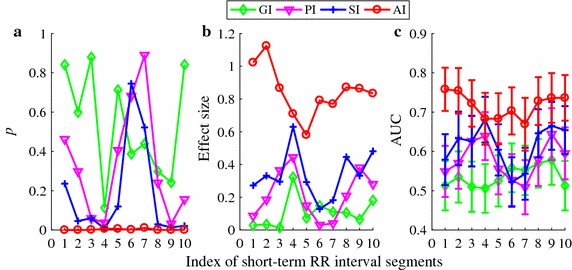
Performance of the four HRA indices (GI, PI, SI, and AI) in differentiating CHF from HEA. All incices were calculated using 10 different short-term (5 min) HRV segments. **a** *p* values; **b** effect size *d*; **c** AUC results (shown the mean and error bar the standard error SE)

We proposed in this study a novel method—area index (AI)—for measuring the heart rate asymmetry (HRA). AI combined information from two aspects (distance and phase angle of each accelerated or decelerated heartbeat to the previous beat), since the change in one of them may not necessarily introduce a change in another, as we shown based on the simulation data (see Additional file 1). Thus, the established HRA measures that were defined based on one feature were not robust like AI (i.e., PI only counted on the number of accelerated or decelerated heartbeats, GI considered only the distance, and SI only took phase angle into account). A shortened data recording may further deteriorate this limitation. Our results from the three different case studies support our hypotheses, showing that AI is a more robust measure of HRA and it performs better than existing indices for assessing HRA from short-term HRV signals. Using the complete recordings, the performance of AI was comparable to SI whereas both PI and GI failed in classifying arrhythmia from normal sinus rhythm as well as in differentiating patients with congestive heart failure to healthy control subjects (Figs. 2, 3). On the other hand, only AI showed acceptable AUC values whereas all the other three methods failed when cutting the completing recording down to short-term series (Figs. 4, 5). The performance of AI was also quite robust when we applied different short-term segments (Figs. 6, 7). In addition, results from our clinical data further supported the findings that for short-term series AI performed better than existing indices (Fig. 8). In this specific case study, the lengths of the heartbeat interval series were not constant; instead, we fixed the lengths of the ECG recordings to 5 min as this is the typical setting in most of the commercialized devices that have built-in short-term HRV analysis module. Even though the actual lengths of the series varied, we expected that AI could still perform better. Our observations seem to support our expectation. In order to better understand how the results change in this different setting, we repeated the analysis protocol c) by using the length of ECG (5-min) as the window length. Still, a 50% overlap between each two windows were set. All the results (Fig. 9) held which indicates quite consistent performance of the proposed AI.

Overall, the results suggest that the distance could be quite sensitive and the information related to asymmetry hidden in distance can be confounded by long-term recordings. That could explain the fact that GI did not show acceptable AUC values for both cases when using long-term recordings and the ROC for GI (which used only the distance information) changed dramatically when using short-term recordings (Figs. 3, 5). However, though it changed, the AUC values were still not acceptable which might suggest that the distance information should be sensitive to mostly the deceleration part which corresponds to the parasympathetic tone [3, 9, 12, 32]. On the other hand, the phase angle might be related more to the acceleration part which corresponds to the sympathetic tone [3, 9, 12, 32], so that it required long data to distinguish healthy from pathology. That is probably why SI worked well when using long-term recordings but failed in short-term analysis. Intriguingly, there seems to be some interaction between the phase angle and distance so that when they were combined (as was done for our prosed AI), the asymmetry was able to be characterized using both the long- and short-term recordings.

In addition, the calculation of phase angle requires a “reference point”—the original point in Poincaré plot. In our study, we have had the minimum RR interval subtracted from each RR interval (see “HRA analyses”) so that the original point has been shifted accordingly. Otherwise, there will be a large “null” area in the Poincaré plot which may decrease the sensitivity of phase angle. There are also other ways to adjust this reference. We are planning to investigate systematically the influence of reference point in the calculation of SI and AI in future studies.

From a clinical point of view, we note that even though the proposed AI shows better suitability to short-term applications and increased robustness compared to existing methods, the AUC values were still relatively low which means the power of AI for the diagnosis of the studied disease is still quite poor. However, as the measurement itself is noninvasive and costless, instead of acting as a diagnostic tool that can find its use in clinical, the AI would have high potential to be a metric for monitoring the ANS balance, which in turn may help evaluate the cardiovascular risk for healthy individuals. Plenty of previous studies have evidenced that the autonomic imbalance can attribute greatly to morbidity and mortality in cardiovascular diseases and have improved predictive power for cardiovascular events than traditional risk factors [35, 38–41]. Our study which aimed at short-term HRV analysis provides the opportunity to incorporate the real-time HRA monitoring to some modern wearable devices, e.g. wrist watch or cell phone with built-in ECG/heart rate measurement module, which will become rather commonplace and will surely be of significant help not only now but also in the future era. Nevertheless, further clinical studies are yet warranted to verify whether our proposed measure is clinically useful for identifying individuals at risk for cardiovascular diseases or as a tool for evaluating different therapeutic interventions.

## Conclusion

A novel area index (AI) for assessing the heart rate asymmetry (HRA) was developed in this study. AI combines the distance and phase angle information of points in the Poincaré plot. Analyses using both the publically available databases and the clinical database suggested better performance of AI in term of classification ability and robustness, which are more obvious when the available heartbeat interval series were short (i.e., 500 intervals or 5 min ECG). AI may potentially offer the opportunity to provide prompt feedback on the change of HRA and even monitor the longtime HRA profile which may be helpful for identifying people of high cardiovascular risk in ambulatory cardiovascular monitoring.

## Additional file

**Additional file 1.** Simulation tests and additional figures.

## Abbreviations

AI: area index
ANS: autonomic nervous system
ARR: arrhythmia
AUC: acceptable area under ROC
CHF: congestive heart failure
ECG: electrocardiogram
GI: Guzik’s index
HEA: healthy
HRA: heart rate asymmetry
HRV: heart rate variability
LI: line of identity
MI: myocardial infarction
NYHA: New York Heart Association
PI: Porta’s index
PVC: premature ventricular contraction
R: R peak
ROC: receiver-operating characteristic curve
RR: distance separating two consecutive R peaks
SA: sinoatrial
SD: standard deviation
SE: standard error
SI: slope index
