# Supplementary material for: Area asymmetry of heart rate variability signal
Source: Biomed Eng Online. 2017 Sep 21;16:112. doi: 10.1186/s12938-017-0402-3 (PMC5607847; doi:10.1186/s12938-017-0402-3)
Supplement: Supplementary file 1 — Additional file 1. Simulation tests and additional figures. [file 12938_2017_402_MOESM1_ESM.docx]

Additional file

**Area asymmetry of heart rate variability signal**

**Chang Yan^1^· Peng Li^1^· Lizhen Ji^1^· Lianke Yao^1^· Chandan Karmakar^2^· Changchun Liu^1,*^**

^1^ School of Control Science and Engineering, Shandong University, Jinan, 250061, China

^2^ School of Information Technology, Deakin University, Burwood, VIC 3125, Australia

^*^ Corresponding Author, E-mail: [changchunliu@sdu.edu.cn](mailto:changchunliu@sdu.edu.cn)

The idea behind our proposed area index (AI) was that we assumed that the asymmetry of Poincaré plot could be reflected in various dimensions, i.e., the distance of points to the line of identity (LI) and the phase angle of points, etc., and that the change in one dimension might not necessarily introduce a change in the other dimension. Here we tried to explain this based on simulated data.

Figure A1 shows an example of two time-series. The time-series were generated using Matlab R2015a. The points below and above LI of Poincaré plot were generated randomly with three restrictions: (1) the number of points above LI is equal to that below LI; (2) the cumulative phase angles of points above LI equals that below LI; (3) the y-value of a specific point equals the x-value of the subsequent point.

Top and bottom panels of Figure A1 show two exemplary time-series of symmetry and asymmetry, respectively. The slope index (SI) values for both time-series are 50% which incorrectly counts the asymmetrical series (bottom one) as symmetrical. However, the AI values for them are 50% and 50.32%, respectively which has corrected the bias of SI for the bottom series.

Our simulation results to some extent suggest certain superiority of the proposed AI algorithm. To be specific, the Porta’s index (PI) only consider the number of points below or above LI, without counting on the relative position of each point [3]. Regarding the Guzik’s index (GI), the Euclidian distance of the point to LI is calculated [1]. Points located in a line which is parallel to LI have same distance which thus introduce a bias to GI. In this case, if the phase angle information is considered, the bias could be corrected. That is basically the idea behind the SI [2]. Still, there is another possibility as we illustrated here that the phase angle information may still be the same but the series is asymmetrical.

Figure A2 shows the receiver-operating curve (ROC) of the four indices for classifying ARR from HEA groups (a), as well as classifying CHF group from HEA group (b) assessed using the complete HRV series (analysis protocols a, see main text). Similarly, acceptable area under ROC (AUC) values were obtained by SI and AI (ARR vs. HEA: 0.86 and 0.76, respectively for SI and AI; CHF vs. HEA: 0.75 and 0.78, respectively) while AUC < 0.65 for PI and GI in both comparisons.

Figure A3 shows the ROC plots of the four indices for classifying ARR from HEA groups (a), as well as classifying CHF group from HEA group (b) using short-term HRV series (analysis protocol b, see main text). Again, acceptable AUC values were only obtained based on AI (AUC = 0.72 and 0.75, respectively, for the two comparisons, AUC < 0.6 for PI, GI and SI in both comparisons).

Figure A4 shows the ROC plots of the four indices for classifying CHF patients from HEA groups using the clinical short-term heartbeat interval data. AUC values of AI is 0.77, and are 0.58, 0.62 and 0.54 for GI, PI and SI, respectively.

**Figure A1** A simulated symmetrical time-series (top left) and a simulated asymmetrical time-series (bottom left) with their Poincaré plots shown on the right panel.

**Figure A2** ROC curves of the four HRA indices – GI, PI, SI, and AI calculated based on the complete HRV recordings – for classifying ARR subject from HEA subject (a), as well as classifying CHF subject from HEA subject (b). The AUC values for them are: (a) 0.57, 0.57, 0.86, 0.76; and (b) 0.62, 0.58, 0.75, 0.78.

**Figure A3** ROC curves of the four HRA indices – GI, PI, SI, and AI calculated based on the 500 intervals of HRV recordings – for classifying ARR subject from HEA subject (a), as well as classifying CHF subject from HEA subject (b). The AUC values for them are: (a) 0.51, 0.50, 0.64, 0.71. (b) 0.55, 0.56, 0.57, 0.74.

**Figure A4** ROC curves of the four HRA indices – GI, PI, SI, and AI calculated based on the 5 min ECG recordings – for classifying CHF subject from HEA subjec. The AUC values for them are: 0.58, 0.62, 0.54, 0.77

**References**

1. Guzik P, Piskorski J, Krauze T, Wykretowicz A, Wysocki H (2006) Heart rate asymmetry by Poincaré plots of RR intervals. Biomedizinische Technik 51:272-275

2. Karmakar CK, Khandoker AH, Palaniswami M (2015) Phase asymmetry of heart rate variability signal. Physiol Meas 36:303-314. doi:10.1088/0967-3334/36/2/303

3. Porta A, Casali KR, Casali AG, Gnecchi-Ruscone T, Tobaldini E, Montano N, Lange S, Geue D, Cysarz D, Van Leeuwen P (2008) Temporal asymmetries of short-term heart period variability are linked to autonomic regulation. American Journal of Physiology-Regulatory, Integrative and Comparative Physiology 295:R550-R557
